# Film-Forming Systems for Dermal Drug Delivery

**DOI:** 10.3390/pharmaceutics13070932

**Published:** 2021-06-23

**Authors:** Larissa Carine Pünnel, Dominique Jasmin Lunter

**Affiliations:** Department of Pharmaceutical Technology, Eberhard Karls University, Auf der Morgenstelle 8, 72076 Tübingen, Germany; larissa-carine.puennel@uni-tuebingen.de

**Keywords:** film-forming system, topical drug delivery, polymeric formulation

## Abstract

Film-forming formulations represent a novel form of sustained release dermatic products. They are applied to the skin as a liquid or semi-solid preparation. By evaporation of the volatile solvent on the skin, the polymer contained in the formulation forms a solid film. Various film-forming formulations were tested for their water and abrasion resistance and compared with conventional semi-solid formulations. Penetration and permeation studies of the formulations indicate a potential utility as transdermal therapeutic systems. They can be used as an alternative to patch systems to administer a variety of drugs in a topical way and may provide sustained release characteristics.

## 1. Introduction

### 1.1. Skin

The skin is the largest organ of the human body [1]. It acts as a protective barrier against external influences such as ultraviolet radiation, chemical and physical insults, attacks by harmful microorganisms, and mechanical irritation. Furthermore, the skin regulates physiological parameters of the body by providing a barrier against water evaporation and temperature loss [2].

Skin diseases such as infections triggered by bacteria or viruses, as well as the immunologically caused chronic skin diseases psoriasis, atopic dermatitis, urticaria, or ichthyosis damage the skin barrier. Physiological functions can no longer be maintained, increasing the risk of further infection. Symptoms such as pain, soreness, and wetness affect the patients’ quality of life [3].

### 1.2. Skin Anatomy

Human skin consists of two layers, the epidermis and the dermis. The epidermis can be divided into four layers, with the stratum corneum being on top, followed by the stratum granulosum, stratum spinosum, and stratum basale [4].

The stratum corneum plays the most decisive part in skin penetration. It consists of dead corneocytes embedded in a crystalline lamellar lipid matrix. In contrast to the living keratinocytes, the already dead corneocytes here have a solid protein envelope that prevents the absorption of substances. This envelope has a hook-like structure which enables the corneocytes to interlock with each other. Between the surfaces of the corneocytes are the corneodesmosomes, proteins that connect the individual corneocytes to each other. The structure of corneocytes, connected via corneodesmosomes, is already formed in deeper layers of the epidermis and is supplemented by further proteins at the transition to the stratum corneum in order to strengthen the mechanical stability of this skin layer [5].

Between the stratum corneum and the stratum granulosum, there are tight junctions that separate the stratum corneum from the lower layers of the epidermis [6].

The stratum granulosum and stratum spinosum mainly protect the lower skin layers from water loss and are the site of differentiation of the corneocytes. The lowest layer of the epidermis is the stratum basale. The keratinocytes are produced from the stem cells contained there [7]. The transit time from their formation in the stratum basale to the stratum corneum is 14 days. The complete renewal cycle takes 28 days [8].

The dermis beneath the epidermis ensures the flexibility of the skin and temperature maintenance of the body. It consists mainly of collagen fibres interspersed with elastic fibres which are surrounded by a matrix of proteoglycans and glycoproteins. Blood vessels, lymphatic channels, and sensory nerves run through the dermis [8]. The dermis and subcutis underneath are not relevant for the penetration of substances for dermal therapy. In systemic therapy with transdermal systems, however, drugs must be able to penetrate to the blood vessels in the dermis and subcutis.

### 1.3. Biochemistry of the Stratum Corneum

The surrounding lipid matrix consists equally of ceramides with a chain length of 16 to 33 carbons, cholesterol derivatives, and free fatty acids, which predominantly have chain lengths of 22 or 24 carbon atoms. The lipids are arranged in a lamellar structure in which the lipophilic parts of the individual components are arranged parallel to one another, and the hydrophilic head groups of the ceramides, cholesterol, and free fatty acids are directed towards one another. The non-covalent interactions of the head groups thus create lipophilic and hydrophilic areas in the stratum corneum from which the lipid bilayer results. Investigations of the composition of the individual components showed that cholesterol derivatives and ceramides have a hexagonal structure in a 1:1 ratio. The addition of free fatty acids results in a shift to orthorhombic packing. The packing density is increased, which leads to the deduction that a balanced composition of the ingredients is essential for an intact skin barrier [7]. Substances can be transported intercellularly, transcellularly, or on a corneodesmosomal pathway through the stratum corneum [4]. The mechanism of the penetration will be addressed in a subsequent part of this review.

### 1.4. Transdermal Transport

**Figure 1:**
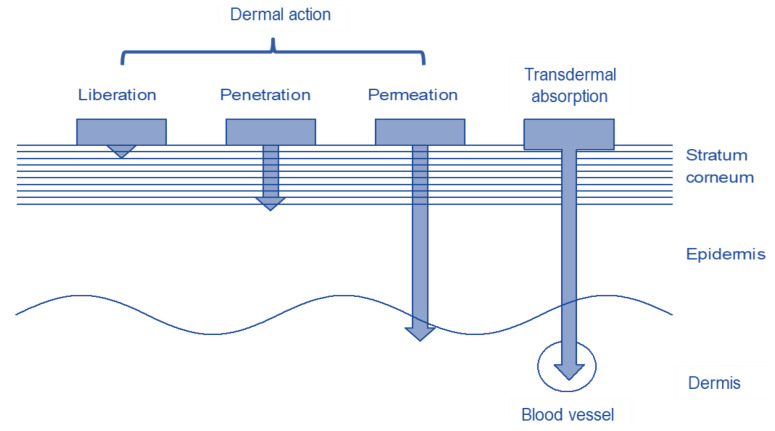
Structure of the skin with the different penetration depths of the dermal and transdermal systems.

Penetration into the stratum corneum is the limiting factor for the amount of drug that can be absorbed [9]. Drugs can pass through the stratum corneum by trans epidermal, trans follicular, or trans glandular routes. Depending on the drug and formulation, they penetrate the skin to different depths as shown in Figure 1 [10]. Whether and how much of a substance penetrates the skin depends on several factors. The properties of the substance to be applied to the skin and the formulation in which it is integrated have the greatest influence on penetration. The condition of the skin, i.e., the skin area and the condition of the skin barrier, which in turn depend on age and individual living conditions, also influences penetration [2].

Three different penetration mechanisms can be distinguished in the penetration of drugs via trans epidermal route. In the transcellular pathway, the drug alternately penetrates the hydrophilic intracellular space, the corneocytes with their protein envelope and the corneodesmosomes, and the lipophilic intercellular space, the lipophilic matrix. This multiple portioning between hydrophilic and lipophilic matrix is very unlikely.

Via the intercellular route, drugs penetrate by crossing the stratum corneum through the lipophilic matrix. Substances that have a predominantly lipophilic character usually penetrate quickly and completely into the lipophilic matrix. As the underlying layers of the dermis are of a less lipophilic character, the penetration of highly lipophilic substances from the stratum corneum into these skin layers is usually slow, so that a reservoir of the substance is formed in the stratum corneum [11]. Drugs with a dominating hydrophilic character can also penetrate via the intercellular route along the hydrated hydrophilic head groups of the stratum corneum lipids. Their penetration by this route may be increased by penetration enhancers or occlusion. Ingredients such as fatty acids, surfactants, and terpenes can be used as penetration enhancers for transdermal transport, while monoglycerides and oxazolidinones are used increasingly for dermal use. Most of them increase the penetration by disruption of the lamellar lipid structure in the stratum corneum. However, the substances used to improve penetration have a disadvantage in that they can permanently disrupt the skin barrier, which leads to skin irritations. The allergenic potential is also increased. These risks can be avoided by combining different enhancers [12]. Another way to improve the penetration of hydrophilic substances is skin occlusion. Formulations with a high lipophilic content reduce the evaporation of water from the skin. The drug can dissolve in the hydrated stratum corneum [13]. The resulting swelling of the skin is often perceived as unpleasant by patients, so other ways of improving penetration should be preferred. Studies have proven that skin occlusion and disruption of the skin barrier can increase the penetration for many hydrophilic drugs, but not for all [7]. Other hydrophilic drugs penetrate via the corneodesmosomal route, which represents the third penetration route of the trans epidermal route. Hydrophilic substances penetrate the stratum corneum along the hydrophilic protein envelope of the corneocytes and the protein-rich corneodesmosomes. Due to the swelling of the corneocytes during occlusion, caused by water retention and disturbance of the skin barrier, these corneodesmosomal bonds can break [6]. Therefore, if a hydrophilic drug penetrates via the corneodesmosomal pathway and not via the intracellular pathway, penetration can be reduced by occlusion or penetration enhancers [6].

In addition to the trans epidermal route, lipophilic drugs in particular penetrate well into deeper skin layers via the trans follicular route. Penetration is rapid as, unlike the trans epidermal route, fewer cell layers must be overcome. However, the amount of active ingredient that penetrates is strongly dependent on the amount and character of the hair glands, so this route depends strongly on individual factors [14].

The glandular route can be neglected, as the majority of the drug is transported out wards by secretion which means that this route cannot be considered for reliable penetration. The penetration pathways described have been researched using healthy skin. In the case of chronic skin diseases such as psoriasis, atopic dermatitis, chronic dermatitis, and ichthyosis, this skin barrier is disturbed, which makes the development of formulations for therapy challenging. The penetration of substances cannot be adequately predicted.

### 1.5. Dermal Systems

Dermal systems are usually designed to only achieve a local effect. They are used for the therapy of acute skin infections and chronic skin diseases. Usually, a liquid or semi-solid formulation is applied at regular intervals. The formulation should be designed in such a way that the active ingredient contained can penetrate into the epidermis and develop its effect. To avoid a systemic effect, penetration into the dermis should be kept to a minimum. Especially in the case of drugs such as antibiotics and immunosuppressants such as cortisone, a sufficiently high therapeutic quantity can be achieved by dermal application without systemically burdening the organism with the drugs. The risk-benefit profile can be influenced positively.

Conventional topical preparations are easily washed or rubbed off. Thus, the treatment is often inadequate as the active ingredient is not present in the epidermis in sufficient and consistent quantities. Using a higher concentration of the drug is not a solution for this drawback, as the concentration fluctuations would only be amplified and the probability of active substance penetrating into the dermis would increase. Formulations exhibiting high substantivity provide a more elegant solution. The substantivity is defined as the capacity of the active substance to be kept at the site of action, on the surface or as a reservoir in the stratum corneum.

Using patches and plasters for dermal treatment of skin diseases may provide an alternative. They create a physical barrier between the formulation and the environment which protects the formulation from erosion. This physical barrier usually leads to occlusion of the skin, which, in addition to the affections of unappealing appearance and negative wearing comfort, is detrimental to patient compliance. In most cases, the patches cannot be cut to the required size, which often makes them unsuitable for individualised therapy. Skin damage can occur when the patches are pulled off, which makes them unsuitable for use in diseases with a disturbed skin barrier. Allergic reactions to the matrix adhesives are another problem, so these systems are not optimal either [15].

### 1.6. Transdermal Systems

In transdermal systems, an active ingredient incorporated into a liquid or semi-solid formulation is applied to the skin, or the drug is delivered via a patch. In contrast to dermal systems, the aim is to deliver the active to the systemic circulation. Compared to other forms of delivery, transdermal systems offer many advantages. They are one way to avoid first pass effect. Food effects or intestinal absorption problems are no issue. Compared to injections or intravenous applications during the administration of drugs, the skin is not injured by needles or incisions [16].

With patches, which are the most common treatment, the sustained release of the drug is usually easy to realise. The drug is embedded in its formulation in the membrane of the patch and may be released through a control membrane or is controlled by diffusion in the adhesive. Patches are usually easy to apply, as they are simply administered at correct time intervals to uninjured, hairless areas of the body surface. When properly applied, the drug release is continuous so that the plasma level is constant. The active ingredient is well protected from external influences in the formulation by the backing layer. In the case of insufficient penetration, in addition to penetration enhancers, systems such as microneedles or iontophoresis can also be used to improve penetration. The disadvantages of patches for transdermal use are the same as in the use of dermal delivery, namely the cosmetic aspect. Currently, mainly opiates and hormones are applied transdermally. However, transdermal application is an option for a lot of drugs with a low therapeutic range and drugs with stability problems [17].

## 2. Film-Forming Systems

As described, the main problem with dermal and transdermal application of drugs via liquid and semi-solid formulations is the washing and rubbing off of the formulations, so that the desired therapeutic effect cannot be achieved. For this reason, very few semi-solid formulations are found in transdermal use. The idea behind the development of film-forming formulations is to develop formulations with increased substantivity against mechanical and water-based influences and improvement of the cosmetic properties, and thus patients’ compliance. As dosage forms, sprays, gels, or emulsions may be formulated [18,19].

**Figure 2:**
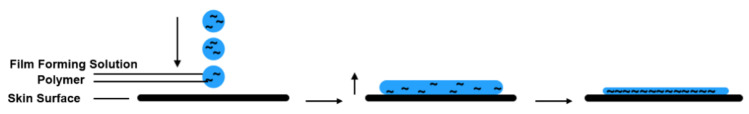
Mechanism of film forming.

**Figure 3:**
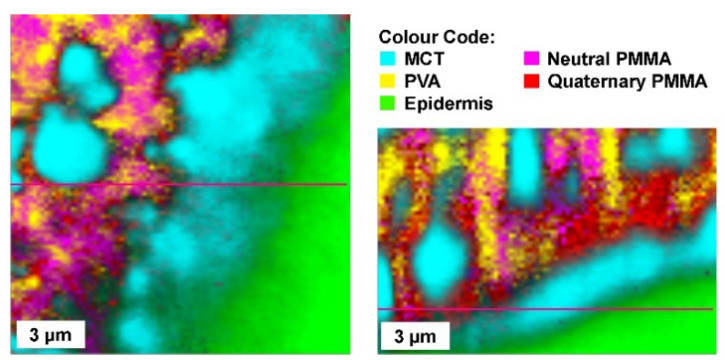
Film-forming emulsion, the oil droplets are embedded in a polymer matrix made of PVA and Eudragit NE (neutral PMMA (Polymethylmethacrylate) and Eudragit RS (quarternatry PMMA) for controlled drug release.

As shown in Figure 2, the formulation is applied evenly to the skin surface. After the application of the solution or gel, the volatile solvent evaporates rapidly and the film-forming polymers interact with one another to form a thin transparent film, which adheres to the skin [20]. Figure 3 shows Raman-microscopic images of a film-forming emulsion applied to the skin. The emulsion is applied in a liquid state and forms a solid film after drying, similar to the film-forming solutions and gels.

In addition to the volatile vehicle and the film-forming agent, the film-forming systems contain the active ingredient and usually a plasticiser and/or penetration enhancer. These non-volatile components ensure that the dry film does not have any brittle properties but adheres to the skin and forms a flexible and non-tacky film after the evaporation of the volatile solvent. The resulting film must be such that it forms a homogenic reservoir of the active ingredient on the skin which has a high substantivity, from which the active ingredient can still penetrate well into the stratum corneum [21]. Due to enhanced substantivity, the formulation can also be used as a transdermal system as the correct dose can be delivered reliably. The formed film must adhere firmly to the skin and must not wash or rub off. The drug can have its reservoir in the formulation itself, and penetrate slowly into the stratum corneum, or pass directly from the formulation into the stratum corneum and form its reservoir there. In the latter case, the formed film serves as protection.

**Figure 4:**
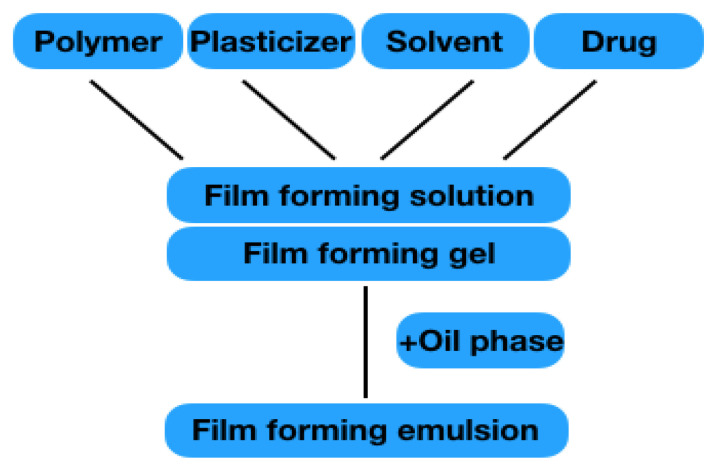
Illustration of different film-forming solution/gel.

This review deals with film-forming formulations containing active ingredients. In the field of semi-solid preparations, gels are listed as film-forming formulations. In the area of liquid preparations, emulsions and solutions, as well as so-called patch-no-patch systems, are discussed. Nano-formulations or formulation for wound healing are not addressed, as dedicated reviews on these topics exist. All formulations mainly consist of the components listed in 2.1.1–2.1.4. The structure is shown in Figure 4. The film formation of the individual formulation results from the polymers it contains. When the solvent evaporates, the polymer chains interact with one another, and are able to form a solid polymer matrix on the skin surface with a high substantivity that forms a reservoir for the drug. The drug penetrates from the film into the skin to exert its effects locally or systemically.

Another type of film formation is the application of an oily formulation that forms an occlusive film on the skin. These formulations are not covered in this review.

### 2.1. Components of Film-Forming Systems

#### 2.1.1. Drug

Regardless of whether the drug is meant to be used in topical or transdermal application, it must be able to penetrate well into the stratum corneum. Drugs with a predominantly lipophilic character usually penetrate the skin better than hydrophilic drugs, as the matrix of the stratum corneum also has a lipophilic character. The distribution coefficient log P is ideally located, intermediate between 1 and 3 [22,23]. The molecular weight plays a decisive role here, as small molecules exhibit a higher velocity of diffusion. The molecular weight should be lower than 1000 Da [22], ideally lower than 500 Da [24]. Another important property of the drug which needs to be taken into consideration during formulation development is the solubility.

If the vehicle of film-forming formulations mainly consists of organic solvent, the drug must be dissolvable in this. Due to the evaporation of the volatile solvent, the drug concentration in the formulation increases. During the evaporation process, the drug must not crystallise, therefore sufficient solubility of the drug in the non-volatile components of the formulation is equally important. Studies show that a formulation with the dissolved drug should have a pH value of 5 to 10 [22]. The pH value of the skin itself is around 5, so the pH value of the formulation should also be in this range to avoid skin irritation during application. Mostly, the optimal pH value for penetration is higher than 7. A pH range of 5–10 is considered as a compromise for both [25,26].

#### 2.1.2. Polymer

The choice of film-forming polymer has the greatest influence on the substantivity of the formulation. Polymers can be used individually or in combination. It is important that they are able to form a flexible, thin, transparent and resistant film. Essentially, a distinction is made between water-soluble and water-insoluble film formers [27]. Water soluble polymers have a hydrophilic character, and most of them are not suitable for substantivity increase in conventional formulations on the skin surface, but ideal for formulations from which the drug quickly penetrates the stratum corneum to form a drug reservoir there. Some, such as methylcellulose, are suitable to increase substantivity of thermo-emulsions by thermo gelation. In film-forming formulations, the aim is to create a drug reservoir in the formulation itself, for which the hydrophilic polymers alone are not suitable.

Water-insoluble polymers form water-resistant films with high substantivity, but are often brittle and inflexible, which makes adhesion to the skin difficult and causes the film-forming formulations to crumble. To increase the uniformity of the film and its flexibility, plasticisers are usually added to the formulation, or the polymer is combined with a water-soluble polymer [20]. Small polymers with a low molecular weight are usually better suited to film-forming systems. The viscosity of the formulation increases during the solvent evaporation process, even more so for polymers with a high molecular weight. Furthermore, smaller polymers with shorter chain lengths can usually arrange themselves better in space, so that the distance for the interaction of the polymer chains is closer to the ideal state for gel formation. After the evaporation of the volatile solvent, the formed film adheres uniformly to the skin, so that no major differences in film thickness result. This is particularly important for the penetration of the active ingredient into the skin, as the concentration gradient between the film and the stratum corneum is thus also constant over a longer period of time on all areas of the skin [28].

Polymers with different properties that have been used for preparation of film-forming systems are mentioned in Table 1. In the future, environmental influences will also become increasingly relevant in the choice of film formers, as most insoluble film formers for modified active ingredient release are considered microplastics, which enter wastewater through precipitation, abrasion, and washing. Therefore, starch, alginate, or cellulose-based formulations should be the focus of the development process in the future [29].

#### 2.1.3. Solvent

In the first instance, the used solvent must be compatible with the skin, even if the skin barrier is disturbed or injured. It should also not irritate the skin during the evaporation process. The film-forming polymer must be well dispersed or dissolved in the solvent. The polymer film should form within less than one minute after the application of the formulation, so the solvent must evaporate quickly at skin surface temperature. At the same time, this must happen uniformly and slowly enough on skin surface that, despite the resulting increase in viscosity of the formulation, the polymers are still sufficiently mobile for interaction and homogenous film forming. Ethanol and isopropanol are particularly suitable solvents for film-forming formulations. Propylene glycol and isopropyl myristate offer the advantage of additional penetration enhancing properties but do not evaporate [21,33,34,35]. In Table 2, a selection of solvents and dispersion agents used for film-forming systems are summarised.

#### 2.1.4. Plasticizer

The main problem during the development of film-forming formulations is the brittle nature of many polymer films. If the film is too rigid, it cannot follow the movements of the skin, especially in application areas such as the elbows [37]. The adherence to skin is then also reduced. A combination of different polymers in one formulation can reduce this problem by taking advantage of different properties of the polymers, but usually the use of plasticizer is necessary [38]. Plasticisers can penetrate between the polymer chains of the film-forming polymer and interact with the functional group. The intermolecular forces between the polymer chains are reduced, meaning that the bonds are weaker and the flexibility of the film is thus increase [37,39]. Studies discovered an ideal plasticizer concentration of 5–20% of the total dry weight [40,41]. A combination of different plasticizers can be also useful. In Table 3, conventional plasticisers used in the development of film-forming formulations are shown, together with the polymers with which they can be combined, according to current studies [37,39,42,43]. The indicated concentration refers to the relative amount with regard to the total dry weight in each case.

### 2.2. Film-Forming Systems on the Market

At present there are several film-forming formulations on the market. These formulations are already approved as medicinal products and are available.

The most successful application of film-forming formulation so far is as wound care products. With the help of the spray plaster, wounds can be completely closed and, if necessary, simultaneously protected against infections by the disinfecting substances contained [45,46]. Some systems containing active ingredients have also received marketing authorisation and are listed in Table 4 with their ingredients. With Lamisil Once^®^, it has succeeded for the first time in developing a formulation for the treatment of athlete’s foot with only one application. The solution is applied and the terbinafine hydrochloride remains as a depot under the film for up to 13 days after application [47]. Axiron^®^ represents the first film-forming formulation for transdermal use containing the hormone testosterone to be sprayed into the axel cavity as a solution and can be used as an alternative to hormone patches. The available spray patch for wound treatment does not contain an active ingredient and is therefore only suitable for wound protection.

Epinamics developed several film-forming sprays with their Liqui-Patch^®^ technology. Formulations with Rivastigmine, Testosterone, Terbinafine, and Vitamin D3 were developed but are not on the market yet [48]. MedPharm and Crescita Therapeutics also succeeded in developing their film-forming systems Medspray^®^ [49] and Durapeel^®^ [50] and making various drugs available dermally, though market approvals have not yet been granted. The systems all contain a polymer and volatile solvent and are listed in Table 5.

It is striking that all film-forming formulations on the market, and also planned ones, contain lipophilic drugs [51]. A possible explanation might be that release of hydrophilic drugs does not need to be slowed down due to their poorer penetration into the stratum corneum. Or, a hydrophilic drug penetrates the skin very poorly or not at all, making the formulation of a film-forming system for dermal or transdermal application obsolete. However, formulations with a high substantivity can remain on the skin for a long time, which allows a slower penetration of the drugs to achieve a therapeutic effect.

**Table 4:** Film-forming formulations on the market [52,53,54,55].

Ingrediencies	Axiron^®^	Lamisil Once^®^	Hansaplast^®^ Sprühpflaster
Formulation	Solution	Solution	Solution
Drug	Testosterone	Terbinafine HCl	-
Polymer	Polyvinylpyrrolidone	Poly(acrylamide-co-isooctylacrylat), Hydroxypropylcellulose	Acrylic copolymer, polyurethane polymer
Solvent	Ethanol 96%, Isopropyl alcohol	Ethanol	Ethanol, water, dimethylether
Plasticizer		Medium chain triglyceride	
other	Octisalate (UV protection)		
Application	Apply once a day to one axilla only	Single application of 2 g solution, drying time 1–2 min	Spray on a thin film of the plaster spray from a distance of 5–10 cm and allow to dry for 1 min.
Company	Eli Lilly	GlaxoSmithKlineConsumer Healthcare	Beiersdorf

**Table 5:** Film-forming systems available on market [48,49,50].

Company	Product	Formulation
Epinamics	Liqui-Patch^®^	Film-forming spray
MedPharm	MedSpray^®^	Film-forming spray
Crescita Therapeutics™	Durapeel	Film-forming emulsion

## 3. Development and Investigation of Film-Forming Systems

Various groups are working on the development of film-forming formulations with different drugs. The topic is especially interesting for the development of sustained-release dermal products. In recent years, various excipients have been compared and tested for their suitability. The development and evaluation of suitable test systems for the comparison of film-forming formulations is indispensable for their development.

The properties of the formulations with regard to drying time, film thickness, film weight, rheological studies, adherence and spreading, stickiness, water vapor permeability, mechanical properties, and film homogeneity must be suitable and compared with one another. The developed formulations should be free of toxic or allergenic ingredients [56]. In case of dermal and transdermal systems, the penetration and permeation of the systems must be investigated [35]. Today, only a few film-forming formulations are on the market. For many drugs for which a dermal or transdermal application option would be useful, film-forming formulations are not yet available. A large number of polymers are still available for the development of new systems. Film-forming systems that have been developed recently will be described in the following chapters.

### 3.1. Film-Forming Solutions

Film-forming solutions are the most common type of film-forming systems. As already described, the solutions mainly consist of a volatile solvent in which film-forming polymers are dissolved or dispersed, together with plasticisers, the drug, and other excipients. The evaporation of the volatile solvent creates a polymer film on skin surface.

Schroeder et al. prepared film-forming solutions by dispersing or dissolving 14 different film-forming polymers with one plasticiser each, triethyl citrate, triacetin, or dibutyl phthalate in ethanol, water, or silicone oil. The film-forming solutions were evaluated for their drying time, cosmetic attractiveness, outward stickiness, integrity on skin, and viscosity. Some dried films were also tested for their mechanical properties such as tensile strength, elongation at break, and water vapor permeability. The developed testing methods are adequate for in vitro investigation. Although, the results cannot be transferred to in vivo, the formulations can still be used as basis for further development of film-forming systems [57].

Edwards et al. investigated polymethacrylate copolymers for their suitability for the transdermal application of drugs using film-forming systems. For this purpose, the group prepared aerosol sprays with methylphenidate and polymethacrylate copolymers and investigated the influence of the solubility of the drug in the preparations on permeation. They were able to establish differential scanning calorimetry for solubility determination. Eudragit E proved to be the most suitable for the development of formulations, as methylphenidate did not crystallise as much here [58].

For further investigation, Gravie-Cook et al. were able to show that the choice of polymer and excipients influence the release from the formulation through interaction with the active ingredient by using Raman spectroscopy and atomic force spectroscopy. Thus, the release of betamethasone from a film-forming solution with hydrophobic polymethacrylate copolymer and medium chain triglycerides was superior to the formulation with hydrophilic polymethacrylate copolymer [59,60].

Misra et al. were one of the first to develop a liquid film-forming solution for the transdermal application of testosterone. For this purpose, testosterone was dissolved in isopropyl alcohol. As film-forming polymers, polyvinyl alcohol and polyvinylpyrrolidone were used in different concentrations. Liquid paraffin and Polysorbate 20 were added. The ex vivo permeation experiments proved a sustained release of the testosterone from the polymer film after a burst release at the beginning. The retarded effect increased with increasing polymer concentration [61]. The approach of transdermal application of hormone derivatives through film-forming solutions was taken up by Schroeder et al., who developed further formulations for the application of ethinyl oestradiol [62].

One application area of film-forming solutions is the dermal application of anti-infectives. Antiseptics and antibiotics need a sufficiently high and constant concentration to effectively reduce or kill bacteria. As the side effect profile increases with higher concentrations, it is particularly advantageous here to transport the active substance to the site of action by the shortest route, and to avoid systematic application in case of dermal or underling tissue infection [63]. To ensure a constant drug concentration, conventional formulations must be applied at regular intervals. Retardation of the dermal system is a promising approach for successful treatment and improved compliance [64].

A film-forming iodophor preoperative skin preparation was developed by CareFusion. Prevail-FX^®^ consists of Povidone-iodine, a complex of polyvinylpyrrolidone and triiodide, which is dissolved in isopropyl alcohol [65]. Investigations with several bacteria proved the antiseptic effect of the formulation. The film formed by the evaporation of the isopropanol remains on the skin long enough to reliably kill bacteria, unlike antiseptic solutions, which do not form a film [65].

For the treatment of dermatological fungal infections, Mori et al. successfully developed a transdermal spray containing voriconazole. The drug was dissolved in a solution of ethanol and acetone. In each formulation, a combination of camphor and menthol was used as penetration enhancers and polyethylene glycol 400 was added. Several combinations of the film formers Eudragit^®^ and Ethyl cellulose were compared to one another. A film-forming spray containing Eudragit^®^ RLPO 10.05% and 5.02% Ethyl cellulose proved to be promising [66].

Nicoli et al. studied the transdermal application of aminoglycosides. They were able to show that the permeation of amikacin in the form of a polymer film is similar to that of conventional gel and is suitable for dermal application. However, in order to make amikacin more readily available in deeper skin layers, procedures such as iontophoresis must be used [67,68].

Another working group has looked at the dermal application of ciprofloxacin for the application to wounds. They created a non-water-soluble film by dissolving ciprofloxacin in an aqueous solution with polyvinylpyrrolidone and further excipients. This transparent film can protect a wound from external influences, and treats the infection at the same time [69]. As there are already spray patches on the market that are used to close wounds, antibiotics such as ciprofloxacin could be integrated into them. The formed film would protect the wound from external influences and at the same time the infection would be treated specifically by the antibiotic depot contained in the film.

Yang et al. developed a film-forming solution for the treatment of MRSA infections in wounds. They developed a formulation of chitosan and polyvinyl alcohol with benzalkonium bromide. The film-forming solution was more suitable for the treatment of MRSA than the comparable aqueous solution with benzalkonium bromide [70].

The topical application of local anaesthetics is another field of application for film-forming systems. On the other hand, a reduction in the concentration of the required drug, to avoid side effects and the prolonged effect at target site, is also the aim here [71]. For topical analgesia, Ranade et al. developed a film-forming spray with the non-steroidal anti-inflammatory drug ropivacaine. The formulations were made of the required drug and various concentrations of Eudragit EPO^®^, which were both dispersed in ethanol and isopropyl alcohol. The analgesic effect of this spray is comparable to that of a conventional lidocaine gel, but it can be applied more easily and less frequently, which increases patient compliance [33].

In addition to conventional patches and other liquid or semi-solid preparations, the patch-no-patch system of Nicoli et al. could be established. They developed solutions of polyvinyl alcohol, Plastoid^®^, polyvinylpyrrolidone, sorbitol, glycerine, lidocaine HCL, and water, which were dried to a transparent film on silicone matrix. The selected skin area is moistened with water before application. Then, the film with silicone paper is placed on the skin area and the silicone paper is removed [72]. The transparent film adheres to the skin surface. The applied patch-no-patch film acts as a matrix-controlled patch which increases permeation, comparable to an aqueous lidocaine solution, without the undesirable side effects such as allergic reactions to the adhesive and cosmetic aspects [73].

To compare different local anaesthetics, they also designed a patch-no-patch with benzocaine 3% and 5% instead of lidocaine, prepared with the same excipients. An in vivo testing in rats showed that the anaesthetic effect of benzocaine was increased compared to a semi-solid formulation, which was confirmed with ex vivo permeation testing. Another part of the study was the comparison of different penetration enhancers in ex vivo permeation. Transcutol^®^, propylene glycol, and isopropyl myristate were used alone or in combination. The analgesic effect and transport of benzocaine could optimized by using a mixture of Transcutol^®^ and propylene glycol [74].

This patch-no-patch system was also developed of Femenía-Font et al. for sumatriptan, not for use as a dermal therapeutic system, but as a transdermal therapeutic system. The film was prepared by hydrating Polyvinyl alcohol in hot water and adding Plastoid^®^ E35H and sorbitol. The sumatriptan succinate solution was incorporated to the mixture. Dependent on the film variant, Transcutol^®^ or Polyethylene glycol 600 was added as penetration enhancer and ethanol as a volatile solvent. The film-forming solution was dried on silicone paper. The permeated amount of sumatriptan from the films was compared with the permeation of sumatriptan succinate solutions. It was found that the effect of penetration enhancers was the same for solution and film. In contrast to the solution, the release of sumatriptan into the skin was more controlled with the film, which is beneficial for the therapy [75]. The group also compared the permeation of Oxybutynin as a film-forming patch versus the conventional patch Oxytrol^®^. The patch-no-patch showed an increased permeation compared to the commercial patch. A lower drug loading and smaller application area can be employed [76].

In developing a patch-no-patch system for the drug furosemide, Patel et al. compared the permeation at different ratios of ethyl cellulose and hydroxypropyl methyl cellulose, and tested the penetration enhancers propylene glycol, isopropyl myristate, and dimethyl sulfoxide. To produce the films, the drug and the polymers were dissolved in methanol and dichloromethane; di-n-butyl phthalate was added as a plasticiser and one of the three penetration enhancers. The solution was dried. The different films were tested for their moisture content and uptake, film-thickness, drug content, and in vitro permeation. The highest flux was determined at a ratio of ethyl cellulose and hydroxypropyl methyl cellulose of 8.5:1.5. Propylene glycol proved to be the best penetration enhancer [31]. Another working group around Amnuaikit also developed a transdermal film-forming patch-no-patch containing Propranolol hydrochloride successfully. As film-forming polymers, ethyl cellulose and polyvinyl alcohol were used in various ratios, always together with the plasticizer dibutyl phthalate. The optimal ratio was found to be twice the amount of ethyl cellulose compared to polyvinyl alcohol. The group also compared different penetration enhancers against each other. By using menthol and cineol as natural substances and propylene glycol as chemical enhancer. Cineole, or a mixture of cineole and propylene glycol, could increase the permeation of propranolol. Film thickness, drug content, and moisture uptake of the films were also investigated [40].

Frederiksen et al. investigated film-forming solutions to achieve the sustained release of betamethasone 17-valerate into the stratum corneum. First, they compared films made of one polymer, which was dissolved or dispersed in ethanol/water solution together with the drug, without using further excipients. As film-forming polymers, hydroxypropyl cellulose, two polymethacrylate copolymers, and a polyacrylate copolymer were used. The release of betamethasone 17-valerat was highest when using hydroxypropyl cellulose as a polymer. The polymethacrylate copolymers and the polyacrylate copolymer have all showed a sustained release. Eudragit^®^ RS performed best. Therefore, hydrophobic polymers are more suitable for reaching sustained release of hydrophobic drugs. On the other hand, the substantivity of the hydrophilic polymer hydroxypropyl cellulose is increased compared to the other polymers. This is an important aspect, especially for topical slow-release formulations. Another topic of the study was the influence of various plasticisers on the permeation of the cortisone derivative. For this purpose, triethyl citrate, tributyl citrate, or dibutyl sebacete were added in equal concentrations to the formulations with the hydroxypropyl cellulose polymer and Eudragit^®^ RS. All plasticizers increased the permeation of the drug independently of the used polymer. The lipophilic plasticizer dibutyl sebacete increased the release the most, followed by tributyl citrate and triethyl citrate [77].

### 3.2. Film-Forming-Gels

Gels are usually transparent and colourless, which makes them cosmetically appealing. Compared to solutions, gels offer the advantage of being easier and more accurate to apply as a semi-solid preparation. The advantage of using film-forming gel preparations instead of film-forming solutions is the easier application of the formulation due the semi-solid character. They are flexible, and mostly show high adherence to skin. Due to the added gelling agent, the polymer matrix is more viscous than comparable liquid preparations, which can be an advantage in the development of sustained release dosage forms. Compared to other semi-solid preparations, gels are particularly suitable for use on mucous membranes [22].

In the field of wound healing, Kin et al. developed film-forming hydrogels with polyvinyl alcohol and polyvinylpyrrolidone as film-forming polymers, propylene glycol, ethanol, and water. The gel showed good adherence to skin and good protection of the wound from external influences. The gel can also serve as a base for the dermal application of drugs [78].

Guo et al. developed an organic-inorganic hybrid gel, which combines the film properties of polyvinyl alcohol, the inhibition of crystallization by using polyvinylpyrrolidone, and the increase in mechanical properties with the use of γ-(glycidyloxypropyl)trimethoxysilane. To form the film-forming gel, Polyvinyl alcohol is dissolved in water and γ-(glycidyloxypropyl)trimethoxysilane is added. The used drug is dissolved in glycerol and polyvinyl pyrrolidone is added. This mixture is integrated into the polymer phase. The viscosity and adhesion to skin of the hybrid gel was investigated, as well as mechanical properties, water vapor permeability, and in vitro release. The gel showed a high adhesive residue on skin while it also exhibited high flexibility in the dry state. The formed film showed a optimal aesthetical appearance by virtue of being thin and transparent, and increased mechanical properties due the integration of γ-(glycidyloxypropyl)trimethoxysilane into the PVA matrix. The use of Polyvinylpyrrolidone and glycerol enhanced the drug permeation. The drug release of Ibuprofen and 5-Fluorouracil was sustained [79]. The permeation from the developed gel should be tested with other drugs in the future.

The working group around Li et al. successfully developed film-forming hydrogels for the transdermal application of tolterodine for the treatment of overactive bladder. The drug, together with triethanolamine was dissolved in water and ethanol and one or a combination of film-forming polymers such as poloxamer, hydroxypropyl cellulose, hydroxypropyl methyl cellulose, and methyl cellulose were added as gelling agent. The permeation and penetration experiments showed a rapid penetration of tolterodine into stratum corneum, in which the drug formed a depot. The bioavailability was high enough for transdermal action and was increased compared to oral bioavailability. The group was able to show that the transdermal application of active substances with film-forming gels is possible in general [80].

### 3.3. Film-Forming Emulsions

Emulsions are liquid preparations and are usually made from an oil phase and a water phase. They are thermodynamically unstable and are therefore usually stabilised with the aid of an emulsifier. Both lipophilic and hydrophilic drugs can be incorporated into the two-phase or multi-phase system. In the case of film-forming emulsions, the used polymers act not only as film-forming agents but also as thermodynamic stabilisers of the emulsion, which may reduce the amount of emulsifier needed to stabilise the emulsion and therefore the risk of skin irritation [81]. Emulsions often show high compliance with the patient, as they are pleasant to apply due to their usually lower viscosity and, in contrast to gels, still have a lipophilic phase without occluding properties [82].

In this type of emulsion, the used polymer is dissolved or dispersed in the volatile solvent and forms the continuous phase. As the solvent evaporates, the viscosity of the polymer phase increases and stabilises the inner phase. The active ingredient is located in the droplets of the dispersed phase and diffuses through the polymer matrix into the skin. This in situ release enables a controlled sustained release of drugs, which is particularly interesting for the development of topical formulations for highly lipophilic drugs with a high affinity to stratum corneum. Due to the localisation of the active ingredient in the dispersed phase, supersaturation and crystallisation of the drug do not occur when the solvent evaporates. This is advantageous, as the thermodynamic properties of the emulsion would change with crystallisation and the emulsion stability could be impaired.

Lunter et al. achieved a sustained release of the active ingredient nonivamide by developing a film-forming emulsion. The compositions of the continuous phase were varied and the emulsions were tested for glass transition temperature, adhesion to skin, elongation, water resistance, and in vitro release. Nonivamide was dissolved in medium chain triglycerides and formed the dispersed phase of the emulsion. For the continuous phase, Eudragit^®^ NE and RS were compared in different ratios, and hydroxypropyl methyl cellulose, polyvinylpyrrolidone, and polyvinyl alcohol were tested against one another as thickeners and polymer surfactants. Triethyl citrate and polysorbate 80 were used as plasticizers in various concentrations. The combination of water-soluble and water-insoluble polymers without using volatile solvent for formulation created the basis for further research in the field of film-forming emulsions [83,84]. The group also developed oil in oil emulsions with nonivamide in castor oil in the disperse phase and polydimethyl siloxanes as the continuous phase and silicone surfactant. The formulations were evaluated for drug content, phase volume, and viscosity of the silicone oil [85]. It has been shown that drug content and phase volume have an influence on the release rate and a sustained release of nonivamide over 12 h was achieved [85].

Heck et al. used nonivamide in castor oil as a disperse phase. They loaded this oil mixture into silica particles and dispersed the particles in a continuous polymer phase of Eudragit^®^ RS 30D and triethyl citrate by using different types of silica particles and preparation methods. The different formulations were tested for their homogeneity, storage stability, substantivity, and ex vivo permeation. Permeation experiments proved the sustained permeation of nonivamide passing the polymer matrix compared to a commercial semi-solid formulation [86]. By loading the lipophilic disperse phase into the silica particles, it was possible to eliminate the need for an emulsifier in the manufacturing of the emulsion. Nevertheless, the formulation was physically stable. This process can be used and further developed in the future to reduce skin irritation [87].

Schmidberger et al. developed thermogelling emulsions with nonivamide in medium-chain triglycerides as dispersed phase and varied the concentrations of methylcellulose as a film former, and the chain lengths of macrogol in the preparations. Other excipients were sodium citrate and water in the continuous phase, and ethanol as volatile solvent. The different formulations were tested for their rheological properties, droplet size, substantivity, and ex vivo penetration in experiments and compared to one another. The formulations showed increased substantivity compared to conventional semi-solid preparations. The high substantivity and the rheological properties were shown to be related. The substantivity increased with increasing methylcellulose concentration [88]. In order to quantify the substantivity, the working group was able to establish experimental set-ups to simulate the clothing-to-formulation and skin-to-formulation contact during ex vivo permeation experiments. They were able to show that the permeated amount of a conventional cream, the thermogelling emulsion, and the film-forming formulation after repeated contact to skin or clothing depends on their respective properties. With the help of this method, the impact of substantivity on permeation of different formulations can be reliably compared and optimised with regard to the concentrations and type of their ingredients [89,90].

Padula et al. developed transdermal films and a microemulsion for the transdermal application of levothyroxine. For the microemulsion, isopropyl myristate and isobutanol were mixed and polysorbate, sorbianmonolaurate, and water were added. The levothyroxine was integrated into microemulsion and remained in the oily outer phase. The permeated amount of levothyroxine from the microemulsion was the same independent of the concentrations. In further steps, the microemulsions were loaded in the transdermal films which improved the skin retention of the levothyroxine which makes the concentration scalable and the use of this microemulsion as a sustained release formulation possible [34].

## 4. Conclusions and Further Prospects

According to previous research findings, film-forming systems have proven to be suitable for use as a delivery form due to their high substantivity and cosmetic attractiveness. Drugs can penetrate from the systems into the skin, which enables dermal and transdermal drug application. The formulations also serve as a depot, which enables a sustained release. While the use of film-forming sprays in the field of wound care is already established and well accepted by patients, film-forming systems are still the exception in the treatment of diseases.

In the field of transdermal application, there is, with the testosterone spray Axiron^®^, already one formulation on the market. For transdermal use, film-forming systems have not yet been able to establish themselves as a therapeutic option against other transdermal systems such as patches. For dermal application, only film-forming solutions for the treatment of topical infections, such as the terbinafine Lamisil Once^®^ spray, have made it to the market; while studies on the treatment of chronic skin diseases were carried out, but no formulation is yet available for patients, neither for daily therapy in the form of cortisone preparations, nor as NSAIDs for acute therapy. The development of therapies for chronic inflammatory skin diseases and topical infections with film-forming formulations could be pursued more vigorously in the future.

Film-forming solutions that are sprayed directly onto the skin create the sensation of a second skin, unlike patch-no-patch systems. On the other hand, precise application and dosing are easier to implement by using patch-no-patch, which are also able to protect the treated skin area and show increased physicochemical stability. Film-forming emulsions also contain a lipophilic phase and can therefore also be used on dry skin areas. Due to their semi-solid character, gels have an advantage in that that they are easier and more precise to apply. All systems are well suited for personalised therapy. The semi-solid and liquid preparations can be produced in individual concentrations and the films can be printed individually.

Furthermore, there are comparatively few developments of film-forming formulations of drugs with hydrophilic character. A focus on the development of such formulations could provide access to further treatment fields.

The formulations can reach out more to the optimal condition of a second skin by optimising the concentration of the individual components and using new ingredients, thus increasing patient compliance. In this context, gels and emulsions can also become more important than the film-forming sprays that have been predominantly established to date, as they are easier to apply.

Another future field of research is the development of lipophilic film-forming systems with oil-soluble film-forming agents. These formulations could be used for chronic inflammatory skin diseases and, in addition to retarded release of the active ingredient and high substantivity, have positive effects on skin properties. This would allow daily therapy with drugs such as cortisone derivatives to be combined with basic or maintenance therapy.

## Figures and Tables

**Table 1:** Polymers for use in film-forming formulations [21,30,31,32].

Polymer	Properties
Polymer	Properties
Carbopol (polyacrylat)	water-soluble, pH sensitive
Chitosan (Poly-D-Glucosamin)	water-soluble at pH < 7
Crosslinked polymer layer XPL	adhesive, elastic
Dermacryl 79 (Carboxylates Ac rylpolymer)	water-insoluble
Ethylcellulose	non-toxic, not irritating, anti-allergic
Eudragit NE (ethylacrylate methylmethacrylate copolymer)	water-insoluble, transparent, elastic, adhesive
Eudragit RL-100 (polymethacrylate polymere)	water-insoluble, transparent, elastic, adhesive
Eudragit RS-100 (polymethacrylate polymere)	water-insoluble, transparent, elastic, adhesive
Eudragit L30D-55 (methacrylate-ethylacrylate-copolymer)	water dispersible at pH 2–3
Hydroxypropyl-beta-cyclodextrin	water-insoluble, increases bioavailability
Hydroxypropylmethylcellulose (HPMC)	water-soluble, non-ionic
KIucel (Hydroxypropyl cellulose)	water-soluble, non-ionic
Macrogol	water-soluble
Methyl cellulose	water-soluble
Poloxamer (polyethylenepolypropylene glycol)	thermoreversible
Plastoid (Butyl methacrylate-methylmethacrylate copolymer)	water-insoluble
Polydimethylsiloxane (PDMS)	water-insoluble, non-toxic
Polyvinyl alcohol (PVA)	water-soluble, adhesive, non-toxic
Polyvinyl pyrrolidine (PVP)	water-soluble, adhesive, increase bioavailability
Quaternary polymethacrylat (QPM)	water-insoluble
Sepineo P600 (acrylamide/sodium acryloldimethyltaurate)	water-insoluble
Silicone	water-soluble, non-occlusive

**Table 2:** Volatile solvents for film-forming formulations [21,36].

Solvent	Properties
Benzyl alcohole	lipophilic, organic solvent
Butanol	organic solvent
Ethanol	organic solvent, volatile, hydrophil
Ethylacetat	organic solvent, lipophilic
Isopropanol	organic solvent, volatile, hydrophilic
Isopropyl myristat	organic solvent, lipophilic, penetration enhancer
Polyethylene glycole	hydrophilic, penetration enhancer
Water	hydrophilic

**Table 3:** Plasticizer for film-forming formulations [37,39,44].

Plasticizer	Properties	Polymer	Concentration in %
Dibutylphthalat	Plasticizer	Eudragit E 100, Ethyl cellulose, Polyvinylpyrollidone, Hydroxypropyl methyl cellulose,	10–40
Glycerol	Plasticizer	Polyvinyl Alcohol, Polyvinylpyrrolidone	10–30
Polyethylenglycol 300 or 400	Plasticizer	Hydroxypropyl cellulose, Eudragit E 100, Carbopol,	5
Polysorbat 80	Non-ionic solubilizer, plasticizer, emulsifier, co-emulsifier	Cellulose Acetate	20–50
Propylene glycol	Polymeric solubilizer, plasticizer	Polyvinyl Alcohol, Polyvinylpyrrolidone, Eudragit L100, Hydroxypropyl methyl cellulose, Ethylcellulose, Carboxy methyl cellulose	5–50
Sorbitol	Plasticizer	Polyvinyl Alcohol, Chitosan	2–20
Triacetin	Versatile water or oil miscible solvent. plasticizer	Eudragit E 100	1.43–5.48
Triethyl citrate	Plasticizer	Hydroxypropyl methyl cellulose, Eudragit RL and NE, Acrylate copolymer, Polyvinylpyrrolidone, Polyvinyl Alcohol	6

## Data Availability

Not applicable.
